# Modern Medicine

**Published:** 1901-09

**Authors:** 


A TREATISE ON FRACTURES AND DISLOCATIONS. For practitioners and students. By Lewis A. Stimson, B. A., M. D., Professor of Surgery in Cornell University Medical College, New York. New (3d) edition. In one octavo volume of 842 pages, with 336 engravings and 32 full-page plates. Cloth, $5.00 net. Leather, $6.00 net. Just ready. Lea Brothers & Co., Philadelphia and New York.

This book has shown much popularity. The second edition, which was a large one, was exhausted in one year. This, the third edition, goes forth with promise of still greater sales, on account of the thoroughness with which it has been revised, and increased size owing to additions in the text and illustrations.

This volume is divided into forty-eight chapters, and it is difficult to say which shows the greatest amount of care and research in its preparation. The introduction is devoted to definitions, statistics, influence of sex, age and season. Two chapters are devoted to pathology and etiology. Then follows a discussion of the early symptoms, diagnosis, repair, clinical causes, complications and remote consequences. The work throughout is practical and will meet with a large sale. T. J. B.

